# Inhalation therapy: current and emerging devices in pediatrics

**DOI:** 10.36416/1806-3756/e20250348

**Published:** 2025-09-22

**Authors:** Maria Paula de Carli Hanel, Júlia Giffoni Krey, Laissa Harumi Furukawa, Marcos Brum, Frederico Friedrich, Leonardo Araújo Pinto

**Affiliations:** 1. Grupo de Pesquisa em Epidemiologia e Genética das Doenças Respiratórias da Infância, Pontifícia Universidade Católica do Rio Grande do Sul - PUCRS - Porto Alegre (RS) Brasil.; 2. Programa de Pós-Graduação em Medicina - Pediatria, Escola de Medicina, Pontifícia Universidade Católica do Rio Grande do Sul - PUCRS - Porto Alegre (RS) Brasil.

## INTRODUCTION

Inhalation therapy remains a cornerstone in the management of pediatric respiratory diseases, providing targeted drug delivery with rapid onset and reduced systemic exposure. However, the pediatric population presents unique challenges related to lung anatomy and physiology, breathing patterns, cognitive development, and treatment adherence, as well as the characteristics of pulmonary disease, which can significantly influence drug deposition and therapeutic outcomes.^1^

Pediatric conditions such as asthma, cystic fibrosis, croup, and recurrent wheezing often require chronic or intermittent inhaled therapies to control symptoms and prevent exacerbations. These respiratory diseases are among the leading causes of morbidity and mortality in childhood, contributing significantly to the high number of pediatric visits to emergency departments.^2^

Given the critical role of inhalation therapy in pediatric respiratory care, a comprehensive understanding of the available delivery systems, their clinical applications, and their limitations are essential to optimizing treatment outcomes. This review aims to examine current modalities of inhalation therapy, discuss emerging technologies, and evaluate their advantages and limitations in the context of pediatric care.

## MODALITIES OF INHALATION THERAPY

Inhalation therapy in children encompasses several modalities, each with distinct advantages and considerations. The primary devices used for delivering inhaled medications include nebulizers, pressurized metered-dose inhalers (pMDIs), dry powder inhalers (DPIs), and soft mist inhalers (SMIs). 

### Regular nebulizers

Jet nebulizers convert liquid medication into a fine mist, which is inhaled through a mask or mouthpiece. They are reserved for the minority of children who cannot be effectively trained to use a spacer device.^3^ ^,^ ^4^

### Ultrasonic/vibrating-mesh nebulizers

These newer nebulizer technologies have gained increasing attention in clinical practice. Ultrasonic nebulizers offer quieter operation and shorter administration times compared with jet devices, but their use is limited by potential drug degradation and incompatibility with certain formulations. In contrast, vibrating-mesh nebulizers combine high efficiency, consistent aerosol generation, shorter administration times, and superior portability, making them especially valuable in pediatric care.

### pMDIs

pMDIs deliver a fixed dose of medication with each actuation. When used with a spacer, they are the preferred delivery system for young children, particularly those ≤ 5 years, with a face mask recommended for infants and toddlers and a mouthpiece for older preschool children.^3^ ^,^ ^4^

### DPIs

DPIs are typically shaped like a tube or disk and include a mouthpiece. Depending on the model, the powder formulation may be preloaded or inserted by the patient, consisting of micronized drug particles alone or blended with larger carrier particles, usually lactose. DPIs are breath-actuated and therefore do not require coordination between actuation and inhalation; however, they demand a rapid and forceful inspiratory maneuver. As a result, they are suitable only for children older than 5-6 years who can generate sufficient inspiratory flow, while younger children usually require alternative delivery systems.^4^

### SMIs

SMIs generate a slow-moving mist that enhances pulmonary deposition. They are propellant-free and are easier to use than pMDIs, but they remain limited for pediatric populations.^4^

The choice of an inhalation device in children must account for developmental stage, coordination ability, and inspiratory capacity. Clinical guidelines emphasize that pMDIs with spacers represent the first-line option in most pediatric age groups, while nebulizers, DPIs, and breath-actuated pMDIs may serve as alternatives depending on the child’s age and clinical condition. Table 1 summarizes age-specific recommendations and suitable alternatives for pediatric patients.

**Table 1:** Recommended inhalation devices by age group in pediatric patients.

Inhaler device	Age group			
	< 3 years	3-4 years	5-7 years	8-11 years
Nebulizer	Possible	Possible for acute asthma or croup		
MDI, small volume spacer & mask	Yes	May transition to no mask		
MDI & spacer; no mask		Possible	Yes	Yes
Dry powder device			Possible	Yes

MDI: metered-dose inhaler. Observation: Pressurized metered-dose inhalers (pMDIs) with spacers are the preferred option across most age groups, while nebulizers, dry powder inhalers, and breath-actuated pMDIs serve as alternatives depending on the child’s age and ability to use the device properly.

## INNOVATIONS IN INHALATION DEVICES AND ADVANCES IN TECHNOLOGY AND DRUG DELIVERY SYSTEMS

Recent innovations in inhalation devices and drug delivery systems for pediatric patients have focused on enhancing the efficiency and usability of DPIs and nebulizers, using through approach specifically tailored for children.

Advances in nebulizer technology, such as vibrating-mesh nebulizers or mesh nebulizers, have markedly improved delivery efficiency. These devices can be adapted to individual patient needs, improving adherence and optimizing pulmonary drug delivery.^5^ Technologies that aimed at improving the use of DPIs by infants include the Infant Air-Jet Dry Powder Aerosol Delivery System (iDP-ADS), which employs a bifurcated two-prong nasal interface and enables consistent monitoring and control of lung pressures and ventilatory parameters, thereby enhancing aerosol delivery.^6^

## CLINICAL AND PRACTICAL ADVANTAGES AND LIMITATIONS OF PEDIATRIC INHALATION THERAPY

Each inhalation device presents distinct advantages and limitations that determine its suitability in pediatric care. Recognizing these differences is essential for individualized tailoring treatment and promoting adherence.

Nebulizers enable aerosol delivery independently of patient coordination or inspiratory effort, making them particularly useful in infants, young children, or patients in respiratory distress. They are frequently employed in emergency settings and for patients with limited cooperation. However, conventional jet nebulizers are associated with prolonged administration times, reduced portability, and the need for meticulous cleaning to prevent microbial contamination.^1^ ^,^ ^4^

pMDIs offer efficient pulmonary deposition, reduced oropharyngeal deposition, and improved safety profiles, especially when used in conjunction with spacers. However, their effectiveness depends on correct inhalation technique, and misuse remains a common barrier to optimal outcomes.^1^ ^,^ ^4^

DPIs are environmentally advantageous and well accepted by older children. However, their effectiveness depends on the generation of adequate inspiratory flow, which may be compromised during acute exacerbations or by children younger than 6 years of age, limiting their applicability in some clinical scenarios.^1^ ^,^ ^4^

SMIs require minimal coordination and no propellants, aligning with environmentally sustainable practices. Despite these advantages, SMIs remain limited in pediatric populations due to their higher cost and restricted availability.^1^ ^,^ ^4^

Overall, inhalation therapy remains a cornerstone in the management of pediatric respiratory diseases, providing targeted, rapid, and minimally invasive drug delivery. However, the effectiveness of this approach depends on the selection and correct use of inhalation devices, which must be tailored to the child’s age, developmental stage, and clinical condition. While pMDIs with spacers remain the mainstay across most pediatric age groups, newer technologies-such as mesh nebulizers and advanced DPI systems-are expanding therapeutic options, particularly for patients with anatomical or functional limitations.

Understanding the strengths and limitations of each device is essential to optimizing treatment, supporting adherence, and improving clinical outcomes. As inhalation technology continues to evolve, integrating these advances into pediatric care will be essential to delivering safe, effective, and individualized respiratory therapy for children.
